# Snoring and obstructive sleep apnoea syndrome among hypertensive Nigerians: Prevalence and clinical correlates

**Published:** 2012-04-19

**Authors:** Adeseye Abiodun Akintunde, Oluyomi Oluseun Okunola, Rotimi Oluyombo, Yussuf Olatunji Oladosu, Oladimeji George Opadijo

**Affiliations:** 1 Department of Medicine, LAUTECH Teaching Hospital, Osogbo, Nigeria

**Keywords:** Snoring, sleep apnoea, hypertension, prevalence, clinical correlates, Nigeria

## Abstract

**Background:**

Obstructive sleep apnoea (OSA) syndrome is a common disorder in the community. Association between hypertension and sleep apnoea and /or snoring has been described. The Berlin questionnaire is a validated instrument that is used to identify individuals who are at risk for OSA. The study aim to describe the prevalence of snoring and OSA among hypertensive subjects in South Western, Nigeria.

**Methods:**

This was a descriptive study conducted at the Cardiology clinic of Ladoke Akintola University of Technology LAUTECH Teaching Hospital, Osogbo, South West Nigeria. One hundred consecutive hypertensive patients were recruited from the clinic. The Berlin questionnaire and the Epworth sleepiness scale (ESS) were used to determine excessive daytime sleepiness and the risk of having OSA. Statistical analysis was done using SPSS 16.0. Data were summarized as means ± S.D and percentages.

**Results:**

The study participants consisted of 40 males (40.0%). The demographic data were similar between both genders except that females had higher mean body mass index than males. The prevalence of snoring was 50.0%. 52% were categorized as being at high risk of having OSA. Snorers were more likely to be older, males and to have a higher fasting blood sugar than non-snorers.96% of snorers reported excessive daytime somnolence as predicted by the ESS score compared to 4% of non snorers. Prevalence of snoring was also higher among overweight and obese hypertensive subjects than normal body mass index hypertensive subjects.

**Conclusion:**

Snoring is common among hypertensive subjects in South Western Nigeria. Clinically suspected OSA was similarly high in prevalence among them. Early identification and management may reduce the cardiovascular risk of hypertensive subjects.

## Background

Obstructive sleep apnoea syndrome (OSAS) is a common disorder in the community [1]. Association between hypertension and sleep apnoea/snoring has been described among many population [2, 3]. The association between sleep related breathing disorders and cardiovascular disease is further stressed by reports of a high prevalence of sleep apnoea among patients with hypertension [3].

Obstructive sleep apnoea syndrome is associated with increased morbidity and mortality. It is characterized by partial or complete collapse of the upper airways during sleep leading to impaired gas exchange and recurrent arousal from sleep. The consequences of OSAS include excessive daytime sleepiness, cognitive dysfunction, impaired work performance, and impairment in health-related quality of life. Observational and experimental evidence suggests that obstructive sleep apnea may contribute to the development of systemic hypertension, [4] cardiovasculardisease, [5] and abnormalities in glucose metabolism [6]. Obstructive sleep apnea is insidious and patients are often unaware of the associated symptoms. Cardinal manifestations include loud snoring, witnessed breathing pauses during sleep, fitful sleep quality, and excessive daytime sleepiness. Early recognition and appropriate therapy can ameliorate the neurobehavioral consequences and may also have favorable effects on cardiovascular health [7]. Habitual snoring is associated with hypertension, cerebrovascular disease and coronary heart disease [8, 9]. The prevalence of snoring among adults varies from different part of the world from 5 to 44% [8, 10, 11]. In a report from Abuja, Nigeria, overall prevalence of snoring among adults was 31% while the prevalence of clinically Suspected Obstructive Sleep Apneas (CSOSA) was 1%, (1.9% in males, 0.5% in females) [11].

There is dearth of information on the pattern of snoring and sleep apnoea among Nigerians with hypertension. The study aimed at describing the frequency of occurrence of snoring and risk of obstructive sleep apnoea among hypertensive subjects and their associated clinical correlates.

## Methods

This was a cross sectional study. It was carried out at the Cardiology unit of the Ladoke Akintola University Teaching Hospital, Osogbo. Osun State Nigeria. One hundred patients being managed for hypertension were recruited consecutively for this study. Hypertension was diagnosed by either a persistent blood pressure >140/90mmHg or the use of antihypertensive medications. Demographic variables such as age, body weight in kilograms, height in meters, waist circumference hip circumference and gender of each participant were documented. Informed consent was taken from each participant. Ethical approval was obtained from the Institutional Ethical Review Board.

The Epworth Sleepiness Scale (ESS) was used to determine excessive day time sleepiness. It is an eight item self administered questionnaire. Possible score ranges were from 0 to 24. For this study, an ESS score of more than 11 was taken to mean Excessive day time sleepiness EDS. The Berlin Questionnaire was used to identify the risk of having clinical obstructive sleep apnoea. The questionnaire consists of 3 categories related to the risk of having sleep apnea. Patients can be classified into High Risk or Low Risk based on their responses to the individual items and their overall scores in the symptom categories. Subjects were categorized as high risk for having OSA if there were 2 or more Categories where the score was positive and low risk if there was only 1 or no Categories where the score was positive [12]. The Berlin questionnaire has been documented to be clinically sensitive and correlates significantly with the presence of OSA among various populations. Clinically Suspected Obstructive Sleep Apneas (CSOSA) was defined in accordance with the 2001 International Classification of Sleep Disorders [13].

Statistical analysis was done using the Statistical Package for Social Sciences SPSS 16.0 (Chicago Ill.) Numerical data were summarized using means and standard deviation while categorical data were summarized using frequencies and percentages. Comparism between groups was done using t-test and Chi square.

## Results

One hundred consecutive hypertensive subjects were recruited for the study. It consisted of 40 males (40.0%) and 60 females (60.0%). The age range was between 39 and 90 years. The mean age was 58.4±11.9 years. There was no significant difference between the mean age of males compared to female participants (57.1±10.8 vs. 59.2±12.6 years, p>0.05 respectively). Similarly systolic blood pressure, diastolic blood pressure, fasting blood sugar and waist-hip-ratio were similar between male and female participants (Table 1). The mean body mass index was significantly higher among females than male hypertensives (28.3±5.5 vs. 24.0±3.9, p<0.05 respectively). The proportion of those with abnormal Epworth sleepiness scale was similar between the male and the female participants. Snoring was commoner among male hypertensive subjects than female hypertensive subjects (55.0% vs. 46.7%, p<0.05 respectively). 96% of snorers reported excessive daytime somnolence as predicted by the ESS score compared to 4% of non snorers.

**Table 1:** Clinical and demographic characteristics of study participants based on gender

Variable	Males (40)	Females (60)	P
**Age (years)**	57.1±10.8	59.2±12.6	0.378
**SBP(mmHg)**	138.0±23.3	137.3±14.8	0.873
**DBP(mmHg)**	82.0±14.8	83.2±10.5	0.661
**FBS(mmol/l)**	5.5±1.3	5.2±1.7	0.539
**ESS**	8.1±1.7	8.1±4.5	0.978
**BMI (kg/m2)**	24.0±3.9	28.3±5.5	0.000*
**WHR**	0.93±0.06	0.92±0.07	0.604
**Frequency of snoring**	22(55.0%)	28(46.7%)	0.043*
**Proportion of high risk of OSA**	21(52.5%)	31(51.7%)	0.783
**Proportion of abnormal ESS score**	13(32.5%)	21(35.0%)	0.879

Key to table: SBP: systolic blood pressure, DBP: diastolic blood pressure, FBS: fasting blood glucose, ESS: Epworth sleepiness score, BMI: body mass index, WHR: waist hip ratio, OSA: obstructive sleep apnoea.

Table 2 shows the demographic parameters between hypertensive subjects with reported history of snoring and non-snorers. The mean age of snorers was significantly higher than non-snorers (59.4±12.1vs. 56.1±11.9, p< 0.05 respectively). Likewise, systolic blood pressure and fasting blood sugar were significantly higher among snorers than non snorers (141.9±21.2, 5.90±1.0 vs. 134.9±14.1, 4.9±1.8, p<0.05 respectively). Mean Epworth sleepiness scale was significantly higher among snorers than non-snorers (8.74±3.9 vs. 7.1±4.7, p<0.05 respectively) reflecting an increased daytime somnolence among snorers. Almost all snorers were found to have a high risk of OSA using the Berlin score compared to only 4% of non snorers.

**Table 2:** Clinical and demographic characteristics of snorers compared to non-snorers

Variable	Snorers (50)	Non snorers (50)	P
Age (years)	59.4±12.1	56.1±11.9	0.041*
SBP(mmHg)	141.9±21.2	134.9±14.1	0.0369*
DBP (mmHg)	82.2±13.4	83.4±10.7	0.778
FBS(mmol/l)	5.90±1.0	4.9±1.8	0.021*
ESS	8.74±3.9	7.1±4.7	0.0429*
BMI (kg/m2)	27.6±5.8	25.6±5.2	0.184
WHR	0.93±0.07	0.91±0.06	0.107
Proportion with ESS high risk of OSA	48 (96.0%)	2(4.0%)	0.000**

SBP: systolic blood pressure, DBP: diastolic blood pressure, FBS: fasting blood glucose, ESS: Epworth sleepiness score, BMI: body mass index, WHR: waist hip ratio, OSA: obstructive sleep apnoea;

* P<0.05

The prevalence of snoring based on age group is shown in Table 3. The highest prevalence of snoring was found in the sixth decade followed by those more than 70 years. Snoring was also found to be commoner among overweight and obese hypertensive subjects as shown in Table 3.

**Table 3:** Prevalence of snoring based on age group and body mass index among study participants

Age groups (n)	High risk of OSA	Snoring present
<40 years (7)	2(28.6%)	2(28.6%)
41-50 (22)	11(50.0%)	12(54.5%)
51-60(34)	20(58.8%)	21(61.8%)
61-70(21)	9(42.9%)	8(38.1%)
>70(16)	10(62.5%)	9(56.3%)
**BMI group**	**High risk of OSA**	**Snoring present**
Normal BMI(46)	22(47.8%)	21(45.7%)
Overweight(23)	14(60.9%)	14(60.9%)
Obesity (31)	16(51.6%)	15(48.3%)

OSA –obstructive sleep apnoea

## Discussion

The study revealed that the prevalence of snoring among hypertensive Nigerians in this study was 50%. It also revealed that almost all the hypertensive snorers reported excessive daytime somnolence as predicted using the Epworth sleepiness scale. Male hypertensive subjects had a significantly higher frequency of snoring than their female counterparts. In men the frequency of snoring was 55% while it was 46.7% among female participants in the study. This is similar to what has been reported among other hypertensive population [14]. The prevalence of snoring among these hypertensive subjects was however greater than that reported among non-hypertensive Nigerians [11]. Also, about 52% of hypertensive subjects were found to be at high risk for having obstructive sleep apnoea in this study. Hypertension is a well documented risk for developing sleep related difficulties such as sleep apnoea, hypopnoea and snoring [2, 3, 5, 7].

Individuals with OSA have been shown to have heightened sympathetic activity due to activation of chemoreceptors leading to peripheral vasoconstriction [15]. OSA also results in the production of important neurohumoral mediators of cardiac and vascular disease [16]. Individuals with OSA have increased production of the potent vasoconstrictor endothelin and impaired endothelial function, which affect vasomotion. OSA has also been associated with systemic inflammation, reflected by increases in C-reactive protein and serum amyloid A levels, which may advance atherosclerosis and is associated with increased cardiovascular risk. Perhaps through its effects on sympathetic activity or because of sleep deprivation, OSA may increase insulin resistance, which promotes cardiovascular risk via multiple pathways [17]. Also, OSA is associated with increased levels of leptin, a hormone secreted by fat cells that is also associated with cardiovascular events.

This study also revealed that hypertensive subjects with positive history of snoring were significantly older than those without history of snoring. Furthermore, body mass index and fasting blood sugar were significantly higher among hypertensive subjects with history of snoring than those without it. This highlights the fact that hypertensive subjects with OSA likely have increased cardiovascular risk. The prevalence of snoring increased with increasing age group with the highest incidence among those in the sixth decade and the very elderly group (>70 years).This agrees with other studies have shown that obstructive sleep apnoea and snoring are directly related to age. With advancing age, sleep-related difficulties become increasingly common and often manifest as subjective complaints of difficulty falling asleep, reduced duration of night-time awakenings, and the amount of night-time sleep obtained [18, 19]. Mechanisms proposed for the age-related increase inprevalence include increased deposition of fat in the parapharyngeal area, lengthening of the softpalate, and changes in body structures surrounding the pharynx [20, 21].

Several epidemiologic studies have described the higher incidence of snoring and sleep apnoea among men compared to their female counterparts. Several explanation account for this: first, women may be less likely to report classical symptoms such as loud snoring, apnoea, nocturnal sorting or gasping [22, 23]. Secondly, differential response of the bed partner to the symptoms of obstructive breathing during sleep may also contribute to the clinical underrecognition of the disorder in women.

This study shows that hypertensive who were overweight or obese were more likely to be having sleep related disorders including snoring than those with normal body mass index. Similarly, a higher percentage of overweight and obese subjects were found to be at a high risk of obstructive sleep apnoea using the Berlin questionnaire. Epidemiologic studies from around the world have consistently shown body weight as the strongest risk factor for obstructive sleep apnea. In the Wisconsin Sleep Cohort study, a one standard deviation difference in body mass index (BMI) was associated with a 4-fold increase in disease prevalence [24]. Other population- and community-based studies conducted in the United States and other countries have confirmed that excess body weight is uniformly associated with a graded increase in prevalence of obstructive sleep apnea [25–27].

## Conclusion

This study therefore shows that snoring is a commonly prevalent associated condition among hypertensive subjects in Osogbo, Nigeria. Similarly many of them were found to be at a high risk of OSA. Snoring and clinically suspected OSA were associated with increasing age, body mass index, fasting blood sugar and systolic blood pressure. Hypertensive subjects with sleep related disorders may therefore have increased cardiovascular risk compared to those without sleep related disorders. Therefore, early identification and management of sleep related disorders such as snoring and obstructive sleep apnoea can further reduce the cardiovascular risk of hypertensive Nigerian subjects.
